# Terahertz Nondestructive Testing with Ultra-Wideband FMCW Radar

**DOI:** 10.3390/s23010187

**Published:** 2022-12-24

**Authors:** Barnabé Carré, Adrien Chopard, Jean-Paul Guillet, Frederic Fauquet, Patrick Mounaix, Pierre Gellie

**Affiliations:** 1 IMS Laboratory UMR CNRS 5218, University of Bordeaux, Bat A31, 351 Cours de la Libération, 33405 Talence, France; 2 Lytid SAS, 10 Rue Alice Domon et Léonie Duquet, 75013 Paris, France

**Keywords:** terahertz imaging, frequency-modulated continuous wave radar, millimeter waves, nondestructive testing

## Abstract

This paper presents the development, performance, integration, and implementation of a 150 GHz FMCW radar based on a homodyne harmonic mixing scheme for noncontact, nondestructive testing. This system offers high-dynamic-range measurement capabilities up to 100 dB and measurement rates up to 7.62 kHz. Such interesting characteristics make this system attractive for imaging applications or contactless sensing. Numerous samples of different materials and geometries were imaged by taking advantage of the radar’s performance. By taking into account the nonionizing capability of the system, new applicative fields such as food industry and pharmaceutical packaging were explored.

## 1. Introduction

For reliability and performance reasons, many industrial problems require access to nondestructive testing (NDT) methods. To complement existing solutions such as X-rays, infrared thermography, or ultrasonic testing, terahertz technology [1] allows noncontact inspection in real time [2], without creating defects [3,4]. Indeed, terahertz waves penetrate a very wide range of materials and are, contrary to X-rays, nonionizing, which makes them harmless. Therefore, as there is no need for protection equipment and safety devices, it is a less tricky solution. Those characteristics are very attractive for the industry because they allow deep control of materials without altering their properties. Unlike ultrasound, the inspection is contactless, making the implementation of the system much simpler. Additionally, the terahertz technique offers a submillimeter resolution, perfect for defect control and adequate for a lot of issues met by industries.

Single-point raster-scanning continuous wave (CW) imaging displays several inherent limitations. Namely, the measurement rates for quasi-continuous wave (QCW) operation are dictated by the response time of the detector, correlated to the use of lock-in synchronous detection. Hence, it abolishes its relevance for industrial purposes. Moreover, coherent sensing grants non-negligible improvements with the acquisition of additional phase information. It is often associated with an enhancement of the measurement dynamic range and recording speed.

The millimeter-wave frequency range can highly benefit from the frequency-modulated continuous wave (FMCW) mode of operation to unlock an additional longitudinal sensing capability [5,6,7]. This work follows the development and the integration of III/V GaAs-based planar Schottky frequency multiplier for the integration of high-power tunable sub-terahertz sources ranging up to 640 GHz by Lytid [8], to evolve towards the design of a fully integrated sub-terahertz FMCW unit. Finally, a working frequency of 150 GHz with a 32 GHz bandwidth was selected. These frequencies feature an excellent compromise between lateral resolution and penetration depth, and they cover a wide range of industrial material issues.

This selection was driven by the well-established capabilities of millimeter-wave FMCW sensors toward nondestructive testing (NDT) topics. Namely, the contactless in-depth inspection capabilities, relatively low implementation cost, compactness, and high measurement rates represent net benefits for such technological candidates when it comes to inspection problems. Such characteristics have been highlighted by the DOTNAC project conclusions [9,10], which additionally featured a net advantage of FMCW approaches for the inspection of a variety of composite materials for aeronautics [6,11] with respect to other well-established NDT techniques. Thanks to the stability and speed of the low-frequency circuit, a high measurement cadency of 7.62 kHz was achieved. Along with a dynamic range up to 100 dB, it provides a very competitive technology for terahertz imaging.

## 2. FMCW Radar Design and Integration

### 2.1. Architecture

**Figure 1:**
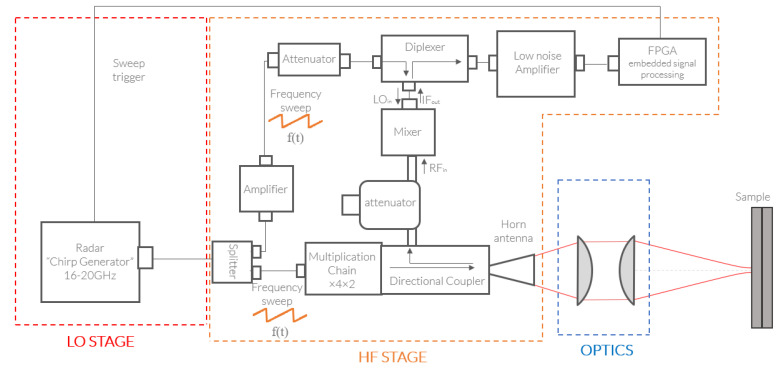
Full FMCW radar architecture diagram, featuring the low-frequency stage, the high-frequency, homodyne detection and emission stage, and the optics.

**Figure 2:**
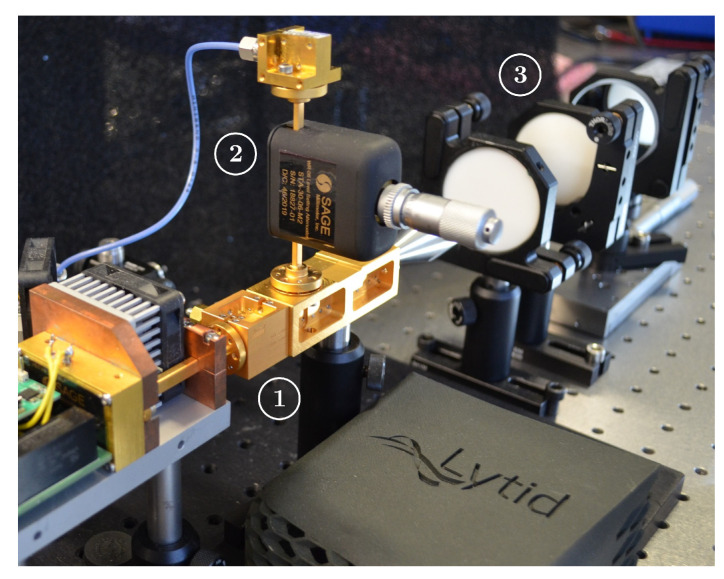
Picture of the FMCW radar transceiver in development. (1) HF multiplication chain, (2) homodyne detection branch, and (3) optics.

As the central piece of the FMCW unit, special attention should be given to the characteristics of the signals provided by the homodyne detection harmonic mixing unit. Namely, in this architecture, on one side the low-frequency local oscillator (LO) is supplied with the swept 16–20 GHz frequency range, and on the other side, the reflected high-frequency (HF) signal is collected as a 128–160 GHz frequency chirp in order to perform the eighth harmonic mixing. To do so, the radar transceiver is split intoseveral primary groups, first with a low-frequency circuit for the generation of this proper LO. It is followed by the upconversion HF chain that will enable the transceiver to reach the submillimeter domain of interest, i.e., the 150 GHz range. It is further completed in reflection configuration by the coupler along with the homodyne detection branch that will allow for FMCW distance-related evaluation. For sensing purposes, beyond the radar transceiver itself, additional beam shaping optics are considered. The complete depiction of each of those stages will be performed in this section and will be supported by their relative performances assessment. To support those technical characterizations, a full architecture diagram depiction is provided in the overview in Figure 1 at the end of this section, and complemented with a photograph of the development integration in Figure 2.

Firstly, to generate the low-frequency sweep, a voltage-controlled oscillator (VCO) integrated in a phase-locked loop (PLL) is used to improve the stability of the generated sweep and to counter nonlinearities. Downstream from the low-frequency sweep generation unit, the direct high-frequency stage is implemented. It features the upconversion frequency multiplication chain based on Schottky multipliers. As displayed in the global depiction of Figure 1, an active amplifier frequency quadrupler is pumped through the previously generated low-frequency signal, leading to the generation of a chirp in the 64–80 GHz frequency range. This emission power is then used for the passive driving of the subsequent self-biased Schottky frequency doubler to reach the 128–160 GHz frequency band of interest. In this monostatic architecture, in foresight of the need for collecting the back-coupled reflected signal, a three-port directional coupler is inserted. This asymmetrical component ensures a proper signal transmission towards the horn antenna, while providing a minimal insertion loss. It then allows for a redirection of the back-reflected signal towards the third output port.

**Figure 3:**
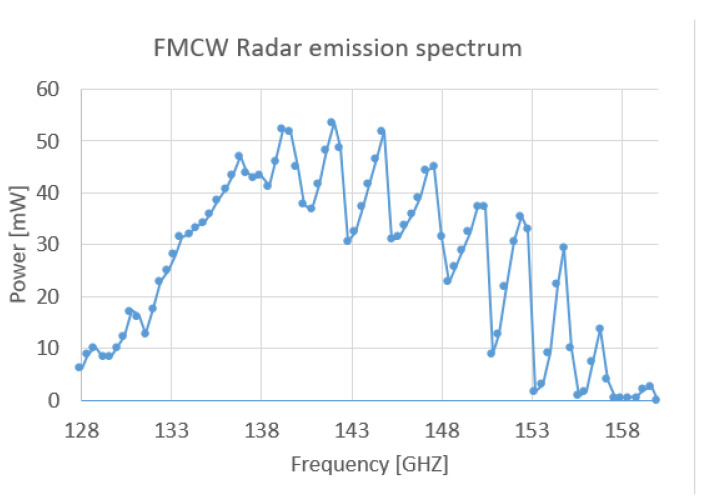
Emission power spectrum of the FMCW radar. The measurement points are the dots. The dots are interpolated by a curve for a better visibility.

With this integration, a WR06 diagonal horn antenna is inserted at the output of the coupler, for free space emission. Accounting for the insertion losses from the directional coupler, the resulting emitted power reaches a peak value of 50 mW, or 17 dbm, with typical power variations induced by waveguide interference cavities that are displayed in the spectral characterization of Figure 3. There are always small impedance mismatches within the waveguides (especially at the connections, or elements inserted into the waveguide geometry) that induce reflections. This can cause standing waves, which will modulate the power of the source. FMCW measurements are not significantly influenced by the fluctuations of the bandwidth power because they are compensated by the normalization process [12].

On the second arm, the LO signal is split and sent to the homodyne detection stage. The mixer receives the LO signal and the reflected RF signal, which are, respectively, two frequency sweeps from 16 GHz to 20 GHz and from 128 GHz to 160 GHz; the RF signal is the eighth harmonic of the LO signal. The harmonic mixer will multiply the two signals, which will lead to an output signal, IF, with the sum of the frequencies $\left(f_{1}+kf_{2}\right)$ of both entry signals and also the difference $\left(f_{1}-kf_{2}\right)$. The difference of frequencies with k=8 is directly linked to the distance of propagation of the RF signal. It informs quantitatively about the position of the scanned sample. Such harmonic homodyne detection requires quite large power levels for the LO. A wideband amplifier is then inserted on this arm right before the homodyne detection, and is followed by an attenuator to fit the LO power level specifications. The value of this supplementary attenuator accounts for the amplifier compression at high input power and the additional insertion losses witnessed from the subsequent SMA (Sub-Miniature Version A) high-frequency cables, as well as the the high-frequency channel of the cascaded diplexer.

Indeed, as the integrated mixer features a single bidirectional SMA for the LO provision and the intermediate frequency (IF) signal retrieval, the integration of a diplexer is required to dissociate those two contributions that remain in very distinct frequency ranges, namely, covering the 16–20 GHz window and below several megahertz, respectively. Such components feature a common port, in our case, directly connected to the mixer LO input/IF output, completed by a high-pass port (16 GHz–20 GHz), from which the LO is provided, and a low-pass port (DC-5 GHz), from which the IF signal is retrieved, as displayed in Figure 1.

### 2.2. Performance and Characterization

Following the detailed depiction of the operation mode of the radar transceiver along with its integrated architecture, a quantitative characterization of its performances is envisaged. Namely, a proper assessment of the lateral and longitudinal resolutions of this unit, with respect to the expected performances, represents ineluctable operational validation factors. The effective sensing dynamic range with respect to the integration time constitutes a central performance factor for any sensing unit. The stability of the FMCW transceiver will be detailed as an integration-oriented characteristic. Finally, as an in-depth sensing unit, it is also essential to measure the longitudinal sensing accuracy and precision.

#### 2.2.1. Lateral and Longitudinal Resolutions

##### Lateral Resolution

**Figure 4:**
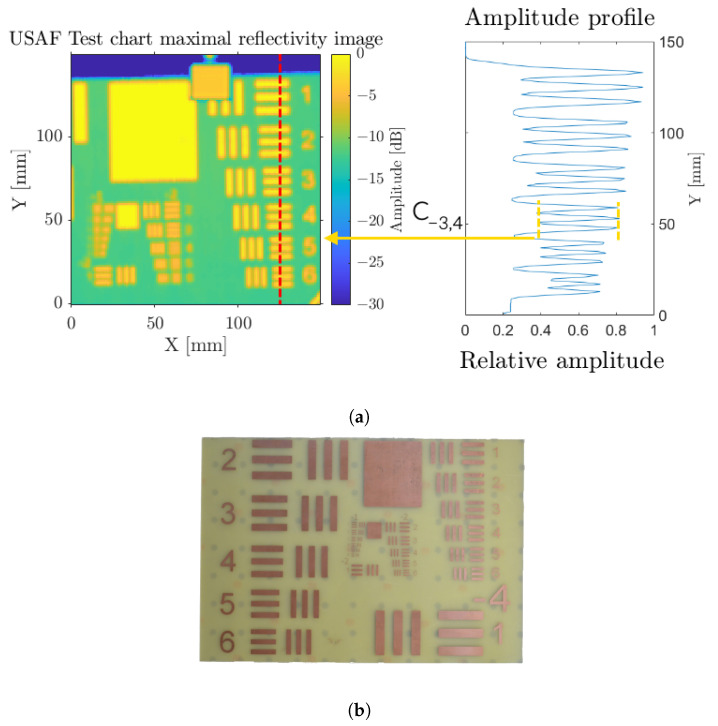
Estimation of the lateral resolution using a 1951-USAF test chart. (**a**) Radar maximal reflectivity image and amplitude profile on a line; (**b**) photography of the USAF test chart.

The lateral resolution remains a criterion of interest for punctual sensing or, in the simplest integration, for imaging purposes when featuring raster scanning. This evaluation was performed using a USAF test chart (Figure 4b), implementing the optical configuration described in Figure 1. As a resolution-oriented investigation, a high numerical aperture (NA) beam focusing was performed using a 2” diameter, f’ = 50 mm aspheric lens, leading to NA = 0.5. From the Gaussian beam model, it ensues that the lateral resolution solely relies on the operation wavelength, 2 mm, and the optical configuration, more specifically the NA.

Figure 4a displays the resulting maximal amplitude image, corresponding to the surface of the used PCB USAF test chart, from which the contrast on each element is assessed. The contrast computations are not performed on logarithmic images but on linear-scale amplitude normalized profiles, such as in the left subplot of Figure 4b. In order to obtain the effective contrast ratio, we should take into account the contrast of the test chart itself. We establish the normalized contrast ratio as ${\hat{C}}_{Grp,El}=\frac{C_{Grp,El}}{C_{t}}$ with the test chart contrast $C_{t}=\frac{1-0.26}{1+0.26}=0.586$. Indeed, for this test chart, the maximal reflectivity value equals a unitary amplitude thanks to the metallized surface, and the low level equals 0.26 (−11.7 dB) relative to the PCB non-null reflectivity. In this configuration, a resolution comprising between 2.8 and 2.5 mm is achieved with measured normalized contrast ratios of ${\hat{C}}_{-3.5}=0.468$ and ${\hat{C}}_{-3.4}=0.575$, respectively. Nevertheless, considering such stable high contrast ratio measurements, a visual resolution of the element 1 group 2 remains practicable for an effective lateral resolution of 2 mm. This difference displays the limit of arbitrary contrast threshold of 0.5, which should depend on several parameters such as the used color scale, the transparency of the substrate, or, as in this case, the use of a logarithmic amplitude scale.

##### Longitudinal Resolution

Besides the lateral resolution, solely linked to the optical configuration and operating wavelength, the longitudinal resolution also represents a parameter of interest for a radar transceiver. This resolution relates to the capability of the transceiver to differentiate two consecutive interfaces along the sensing optical axis and is solely tied to the transceiver achievable bandwidth (see Equation (1)).

(1) $$\delta_{d}=\frac{c_{0}}{2nBW}$$

where $\delta_{d}$ is the longitudinal resolution, $c_{0}$ is the speed of light in vacuum, *n* is the index of the material, and BW is the radar’s bandwidth. Obviously, this quantity will be limited by the numerical frequency resolution. In the data acquisition process, as long as the signal is recorded over a full unidirectional sweep, the resulting numerical frequency resolution after the Fourier transform operation will remain constant as $\delta_{f}=\frac{F_{s}}{N_{s}}$ [Hz], regardless of the sampling frequency, $F_{s}$. The numerical distance resolution can be defined as $\delta_{d}=d_{k+1}-d_{k}$ [m], with *k* and *k* + 1 as the subsequent numerically spaced frequency or distance bins.

(2) $$d_{i}=\frac{c_{0}}{2n}\tau_{i}=\frac{c_{0}f_{IF_{i}}}{2n}\frac{T_{s}}{BW}$$

Considering, then, a numerical depiction of the frequency as $f_{IF_{i}}=k\delta_{f}$, and the frequency to distance conversion of Equation (2), one can deduce, as follows, that the distance resolution is directly linked to the frequency resolution:

(3) $$\delta_{d}=\frac{c_{0}\left(\left(k+1\right)-k\right)\delta_{f}}{2n}\frac{T_{s}}{BW}=\frac{c_{0}\delta_{f}}{2n}\frac{T_{s}}{BW}$$

From there, since $\delta_{f}=\frac{F_{s}}{N_{s}}$ and $\frac{F_{s}T_{s}}{N_{s}}=1$, one can easily return to the initial range resolution definition of Equation (1), solely tied to the effective chirp bandwidth. The numerical longitudinal resolution is then equal to the longitudinal resolution of the radar, the expected 4.6 mm according to the 32 GHz bandwidth.

Now, considering windowing in Table 1 and the zero-padding operation detailed in Longitudinal Precision Section, a frequency numerical resolution enhancement will be artificially accessed. The numerical frequency bin resolution will then be reduced by a factor equal to the $N_{ZP}$ zero-padding factor as $\frac{\delta_{f}}{N_{ZP}}$, hence leading to a distance numerical resolution of $\frac{\delta_{d}}{N_{ZP}}$. A typical factor $N_{ZP}=64$ in this implementation then leads to a numerical distance resolution of 71 μm. This improvement only applies to the numerical resolution, hence the spacing between two distinct frequencies; however, the radar longitudinal resolution obviously does not follow such improvement when enduring the zero-padding operation, due to the convolution of its spectral profile by the Fourier transform of the chosen apodization profile (i.e., rectangular window, Hamming, Hann, or Blackman windows, etc.). As detailed in Table 1, the main lobe width is related to the effective −3 dB longitudinal resolution, through the following equation, directly reformulated from Equation (3):

(4) $$\delta_{d}=\frac{c_{0}\delta_{f_{-3dB}}}{2n}\frac{T_{s}}{BW}$$

In the case where no apodization is performed, with the intrinsic use of rectangular windows, a squared sinus cardinal $sinc^{2}$ function will be convoluted with the expected Dirac spectral response. A 0.87 × $\delta_{f_{-3dB}}$ main peak width will be reached, leading to an achievable longitudinal resolution of 4 mm. Such a measurement will nevertheless be highly limited by the −13 dB sidelobes. Respectively, 5.9 mm, 6.5 mm, and 7.4 mm effective resolutions will be accessible when integrating the Hamming, Hann, and Blackman apodization windows. A trade-off between achievable measurement dynamic range, limited by the sidelobes amplitude, and longitudinal resolution then directly emerges from the values given in Table 1.

#### 2.2.2. Dynamic Range

**Figure 5:**
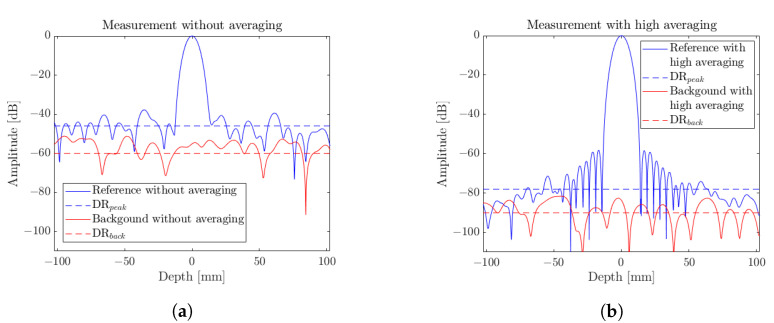
FMCW radar amplitudes spectrum for normalized references and background measurements (**a**) when no averaging is considered for high-cadence measurements, and (**b**) at high averaging levels, for high-dynamic-range sensing equivalent to a 1 min equivalent integration time.

The measurement of the dynamic range (DR) granted by a system remains a central characteristic. This quantity solely relies on signal amplitude considerations. Obviously, an averaging operation over several sensing frequency chirps will have a tendency to improve the effective signal-to-noise ratio (SNR) on this given signal. This impact is directly perceptible in Figure 5, which displays amplitude signals in the absence of any numerical mean operation, hence allowing for the highest measurement rate in Figure 5a, and in the case of high levels of averaging in Figure 5b, targeted for high-dynamic-range measurements. As a prior step, a signal normalization is performed at the calibration of the radar with a temporal normalization transfer function [12]. It consists of taking one measurement over a perfect reflecting object at the optimal imaging plan, a reference, and another one taken with nothing on the sensing area, a background. Two types of numerical averaging are considered. At first, an averaging over the recorded normalization reference and background signals is performed. This data recording is considered only once in the calibration step thanks to the high stability of the sensor, as detailed in Section 2.2.3. Secondly, an averaging over the measured sample, either before or after the normalization, will highly impact the achievable dynamic range, however impacting the actual sensing acquisition rate of the radar.

Additionally, two dynamic range configurations are observed from Figure 5. A global measurement dynamic range, DR${}_{back}$, that best suits the effective definition of the dynamic range, depicts the ratio between the smallest reflectivity detectable with respect to the unitary reflectivity reference. The assessment of this quantity is performed on a background configuration measurement. In addition, a DR on reference, DR${}_{peak}$, is established and will be estimated as the smallest reflectivity signal detectable in the presence of a highly reflective element. The evaluation of this quantity is, hence, performed on a reference measurement. In each of those cases, their values are assessed as the mean noise value over a window of interest. Precisely, a ±10 cm longitudinal measurement range is considered. For DR${}_{back}$, the mean value over the whole range is considered to assess the noise level. In the case of DR${}_{peak}$, the central lobe of the peak is omitted. DR${}_{peak}$ is then attributed as a mean noise level surrounding the central lobe.

**Figure 6:**
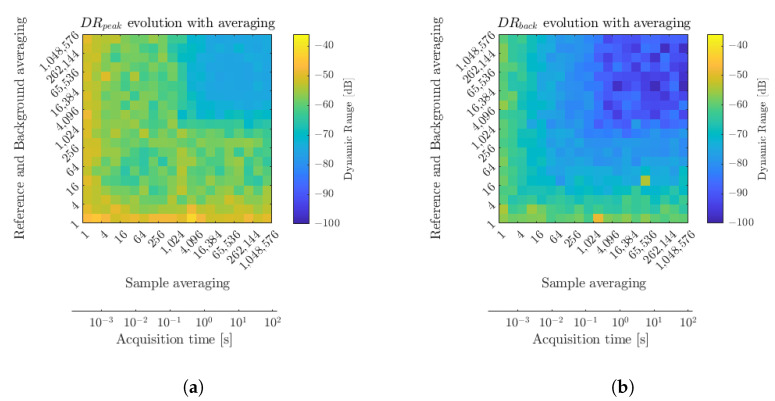
Dynamic range evolution of DR${}_{peak}$ (**a**) and DR${}_{back}$ (**b**) with sample averaging and reference and background averaging, adding a parallel between sample averaging and acquisition time.

An evaluation of those two quantities with respect to the imputed averaging levels was explored and the results are displayed in Figure 6. At first glance, one can notice that, as expected, the DRs feature an improvement tendency with respect to the two averaging operations. The effective DR, DR${}_{back}$, ranges from 60 dB at a full speed of 7.62 kHz over a 125 μs symmetrical chirp, down to over 95 dB when considering integration times above several seconds, for averaging levels over 65,536. An optimal value of 98 dB was recorded for an 8.5 s integration time and 16,384 averaging over the reference and background measurements.

The DR over peak, DR${}_{peak}$, on the other hand, reaches a value of 46 dB in the absence of averaging and features a saturation around 75 dB as the Blackman window profiles then display predominant limiting sidelobes. This saturation occurs above an averaging factor of 4096, hence a measurement cadence of 1.9 Hz, from the moment where DR${}_{peak}$ reaches the sidelobe levels at −60 dB. This saturation is effectively displayed in Figure 5b, with the appearance of the Blackman pattern. The offset between the two dynamic ranges comes from the additional noise components introduced by the normalization procedure in the presence of a highly reflective sample. In imaging conditions, this could relate to a longitudinal glare noise. As expected, a clear trade-off between achievable measurement dynamic range and measurement rate is drawn. Those measurements can then be provided as abacus data for such considerations. The optimal windowing apodization profile can then also be assessed according to the achievable DR${}_{peak}$. Indeed, thinner main peak profiles are considered for low-dynamic-range measurements (see Table 1). In contrast, long-integration high signal-to-noise ratio (SNR) measurements depend on sidelobe minimization oriented windowing profiles, for example, the Nuttall window [13] that features sidelobes below 95 dB at the expense of a larger main lobe.

The state-of-the-art published III/V FMCW radar transceivers in the 150 GHz band feature a 40 dB dynamic range, for an acquisition time of 100 μs, 60 dB at acquisition time of 100 ms, and 70 dB for a 10 ms acquisition rate. Those values were extracted from the DOTNAC project final report [9,10,14]. For comparative purposes, at similar integration times, of 100 μs, 10 ms, and 100 ms, and practicable background and reference averaging of 2048, the developed transceiver features respective dynamic ranges DR${}_{back}$ of over 57 dB, 77 dB, and 85 dB. Further III/V FMCW radar sources, operating at various frequencies, demonstrated measurement dynamic ranges around 60 dB without integration time specifications. Several other systems are highlighted, with [15] operating at 675 GHz, leading to 63 dB with chirp times of 100 μs and unspecified amount of averaging, 60 dB for a transceiver operating at 100 GHz [12] over 5 ms acquisition rate, or, again, 60 dB operating at 200 GHz [16]. All these values have been summarized in the Table 2. The dynamic range performances nevertheless remain quite often access-limited when it comes to commercially available systems, hence the absence of more recent systems’ capabilities in this comparative study.

The lack of such quantitative performance assessments is also witnessed in the recent development of silicon-based FMCW transceivers. In the case of the silicon-radar transceiver, besides the range accuracy [17], and electronic-integration-oriented developments [18,19,20], no short-range-sensing performances oriented studies have been performed. From lab development, typical dynamic ranges up to 50 dB are achieved, depending on the implementation, with a measurement rate of several hertz (up to 20 Hz). This can partially be explained by the targeted design of this component, better suited to longer measurement range considerations where 70 dB dynamic ranges are obtained. Besides their typically reduced achievable bandwidth, and hence longitudinal resolution, their considerably lower emission power also remains an intrinsic limitation.

Lastly, the analysis of the FMCW radar transceiver DR detailed in this section was performed in a focused point configuration, hence related to the high emission power of the transceiver, requiring the insertion of a 16 dB attenuation for the safe operation of the frequency mixer. The additional 16 dB of potentially available power could then be accounted for in the performances of the radar in specific configurations such as when investigating low-reflectivity targets. The large available emission power, in contrast with Si-based radar transceivers, presents a net advantage for far-field open beam synthetic aperture radar (SAR) [21,22] investigations where most of the emitted power is not back-coupled toward the transceiver, preventing the mixer from enduring any damage.

#### 2.2.3. Stability

Besides the effective dynamic range, stability performances remain of strong interest for system integration and operation-oriented problems. Namely, this characterization should assess the validity time of the normalization procedure [12] and highlight the requirement of an eventual periodic recalibration of the system. Indeed, a slight evolution of the reminiscent nonlinearities of the transceiver or spectral characteristics for the LO and/or source spectrum should impact the measurements normalization reference, ${\tilde{S}}_{IF_{ref}}\left(t\right)$, and background, ${\tilde{S}}_{IF_{back}}\left(t\right)$. A regular update of those signals should then be considered if such variations were to be endured.

**Figure 7:**
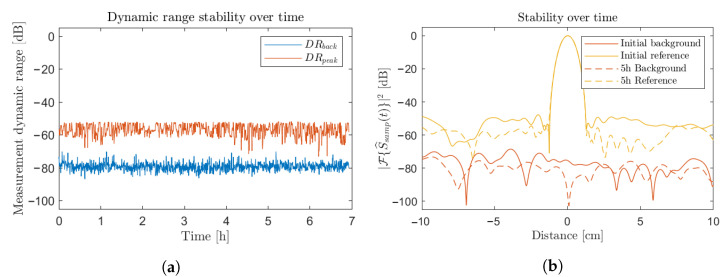
(**a**) Dynamic range stability over time; (**b**) reference and background signals taken right after the calibration and after 5 h of operation.

Figure 7a displays the evolution of the background DR, DR${}_{back}$, and the DR on reference, DR${}_{peak}$, as a function of time over a long operating period. The considered reference and background normalization signals were taken right at the beginning of this measurement period after several hours of operation of the radar for optimal thermal settling purposes. Averaging levels of 256 were considered so as to fit a typical imaging configuration. Those extractions were performed by consistently measuring two signals in a reference and background configuration, respectively, over several hours in a controlled environment. No significant drifts or degradation of the respective dynamic ranges were observed over a measurement period of 7 h. Alongside this, arbitrary signals taken, respectively, right after the calibration and after 5 h of operation are displayed in Figure 7b. No noticeable peak distortion or significant noise level rise are observed. Figure 7b nevertheless features a DR${}_{peak}$ variation of several dB when considering a distance of 3 cm over the initial reference and the 5 h reference measurements. This discrepancy emerges from the higher variation level witnessed over the DR${}_{peak}$ evolution displayed in Figure 7a. It emanates from the sensitivity of the raw signal normalization procedure to subtle target displacements or vibrations. In detail, featuring such a stability up to several hours, no additional recalibration is required when operating in a controlled environment, especially when regulated in temperature. Ultimately, for optimal performance, a settling time is nevertheless required upon the transceiver startup to allow for each component to reach thermal stability.

Indeed, the effective normalization transfer function [12] might be ever so slightly impacted by the characteristic of each component in the transceiver architecture. Namely, a thermal stabilization of each element at startup is a significant factor, hence taking into account the settling time as a parameter of importance. Considering a normalization signal taken after several hours of operation, a 10 to 15 dB settled improvement is noticed on DR${}_{back}$, with respect to the initial signal. A total stabilization of 1 hour of operation prior to the recording of the normalization signals would grant optimal performances. Nevertheless, the transceiver operation right after startup still features ideal performances when ensuring regular recalibration over the first 15 to 30 min of operation.

#### 2.2.4. Longitudinal Sensing Precision and Accuracy

##### Longitudinal Precision

**Figure 8:**
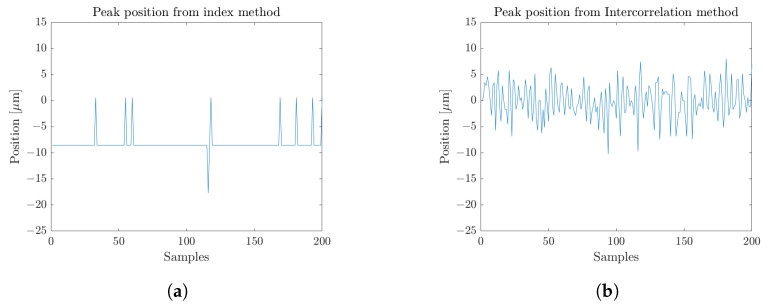
Main peak position over 200 consecutive measurements, on a fixed reflective sample, retrieved through (**a**) the index of the maximum of reflectivity with $N_{ZP}$ = 512, or (**b**) the maximum of an apodization window intercorrelation.

Unlike the longitudinal resolution that refers to the capability of the transceiver to discern two distinct targets, the longitudinal precision depicts the stability performances of the radar to retrieve the longitudinal position of a given reflective interface. It would then be equivalent to the frequency jitter witnessed on the main signal peak. At first glance, the longitudinal precision could be considered as the stability of the maximum of reflectivity of an interface. During the normalization data processing step, the zero-padding method is applied. It consists of adding zero value point in the temporal domain to improve the numerical range resolution in the frequency domain. Nevertheless, it still leads to a finite distance resolution. The higher the zero-padding factor, *N*${}_{ZP}$, the finer the numerical distance resolution. Figure 8a demonstrates that even at high zero-padding factor, the achieved numerical resolution remains insufficient, especially for stable high averaging sensing. Typically, for such type of resolution-oriented measurements, the integrated *N*${}_{ZP}$ = 512 factor leads to a numerical resolution of $\delta_{f_{N_{ZP}}}=\frac{\delta_{f}}{512}=30$ Hz, hence a longitudinal numerical resolution equivalent to a distance of 9 μm. Further subsampling could be performed but would therefore require additional unnecessary processing resources. To overcome this longitudinal resolution limitation, another approach to assess the peak stability, or precision, lies in the consideration of the peak in its entirety, and not only considering the maximal point. As the peak profile is well established by the apodization window, an intercorrelation between the measurement and the ideal peak profile will return a maximal amplitude at the position where both are coincident. By interpolating the numerical measurement, this estimation is also extended below the longitudinal numerical resolution of $\frac{\delta_{f}}{N_{ZP}}$. Operating this algorithmic approach over subsequent measurements will allow to precisely assess the position of the peak over time, as displayed in Figure 8b. The consideration of the root mean square (RMS) of the position evolution then leads to the longitudinal precision.

**Figure 9:**
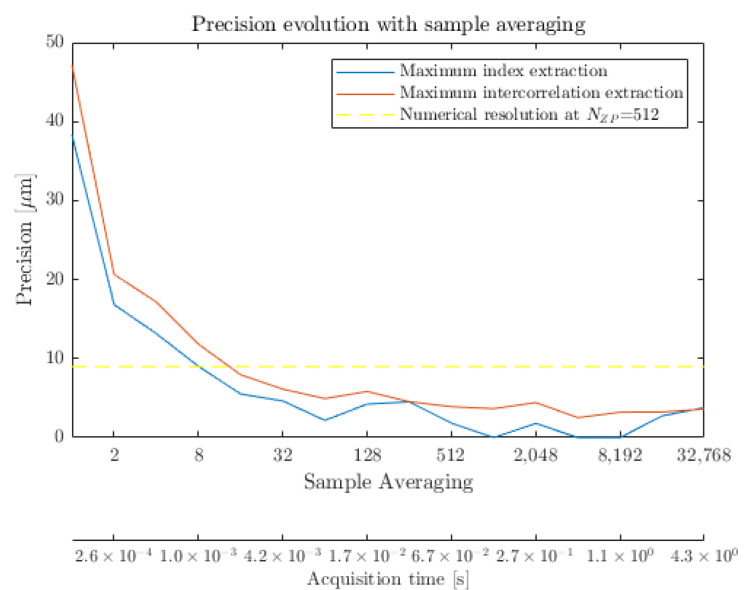
Evolution of the longitudinal precision with respect to the sample measurement averaging.

Obviously, a major parameter for this characteristic is the averaging level of the sample measurement. Figure 9 details the evolution of such assessed longitudinal precision with respect to the integration time. A Blackman window Table 1 was integrated with the previously mentioned zero-padding factor of $N_{ZP}=512$. The numerical resolution of 9 μm then prevents the maximal reflectivity index approach from providing relevant information below this value, as displayed in Figure 8a. A global undervaluation of the precision is then highlighted with this approach, as depicted in Figure 9.

Considering the intercorrelation extractions, the measurement precision then ranges from 50 μm for single-shot measurements down to 3.6 μm when considering a 4.2 s integration time; hence, considering an object placed at roughly 50 cm, there is less than 0.001% positioning instability. Such fluctuations are tied to a variety of factors, either linked to the transceiver itself or its environment. Namely, mechanical vibrations of the workstation are recorded as a strong impacting factor for this measurement. Additionally, thermal effect can affect the electronic components among the transceiver and might also induce a subtle change in the refractive index of the propagation media, on the order of several parts in a million (1 × 10${}^{-6}$) for a 1 °C temperature variation [23].

##### Longitudinal Accuracy

**Figure 10:**
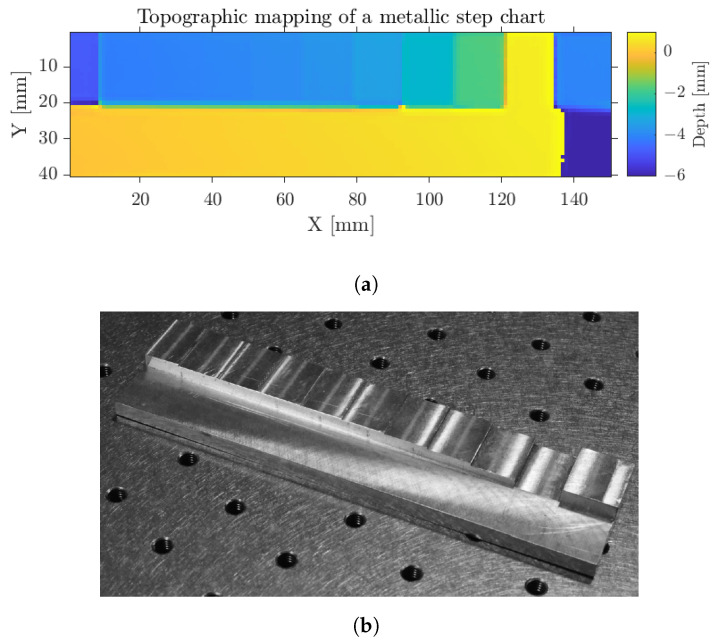
(**a**) Topographic mapping of a metallic step chart and (**b**) photography of the sample.

As depicted in Figure 8a,b, the slight variation of central value between the two measurements brings up the difference between precision and accuracy. The accuracy relates to the effective position assessment, hence the capability of the transceiver to procure correct values, while the precision details the spreading around the mean returned value. From the normalization algorithmic process [12], the position output by the radar is given with respect to a reference plane. An absolute accuracy evaluation is then impracticable. However, a relative extraction was carried out with the use of a step metallic target [24], displayed in Figure 10. The sample is composed of a flat reference surface for post-processing alignment purposes and subsequent steps of different heights given in column 1 of Table 3.

Manufacturing mechanical uncertainty in the order of ±10 μm, from the numerical positioning tool, should additionally be highlighted. A full scan of the sample was performed and the topographic image, resulting from the previously detailed intercorrelation extraction approach, is given in Figure 10. The extracted step size lies on the differential depth measurement performed on the averaged central region of each step to avoid any edge effects. Considering a 10% relative error as the resolving threshold [24,25], in accordance with the ±10 μm mechanical imprecision, a relative longitudinal accuracy of 110 μm step is then reached, as depicted in Table 3. A consistent measurement is also achieved for a step of 25 μm, even though below 50 μm step sizes, the 10 μm mechanical machining uncertainty already induces a 20% relative error. Due to the uncertainty, measurements under 110 μm are not reliable.

Such accuracy limitation can emerge from two main considerations. The integration of the noisy components around the main measurement peak in the intercorrelation extraction can lead to slight peak shift. A peak distortion can also be induced by a reminiscent nonlinearity among the frequency chirp; hence, the intercorrelation with an ideal Blackman window could lead to this considered shift with respect to the maximum peak position. The assessment of the longitudinal accuracy and precision were performed only through a sole consideration of the amplitude profile. A whole complex signal consideration, especially accounting for the embedded phase, should lead to higher precision levels.

## 3. FMCW Imaging

Initially intended as one of the intrinsic applications for the FMCW technique, thanks to the longitudinal sensing availability, three-dimensional volumetric testing grants a perfect demonstration of the inspection capabilities embedded with such approaches. Accounting for the performances of the previously featured 150 GHz transceiver, detailed in Section 2.2, namely, the high-dynamic-range and millimeter-level resolutions, a variety of challenges are tackled, providing that the inspected material remains suitable. Specifically, in the absence of metallic parts, a broad spectrum of dielectric materials, ranging over polymers, textiles, ceramics, plasters, glass-fiber-based composites, etc., are appropriate for the detection of breaking or stress marks, foreign material inclusions, delaminations, density variations, humidity traces, or, more generally, health and structural monitoring.

**Figure 11:**
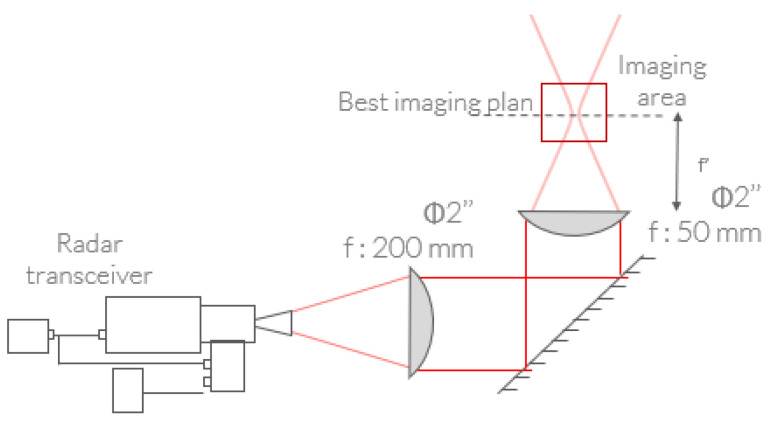
Typical optical setup for FMCW radar nondestructive imaging in far-field configuration.

For such proof-of-work demonstrations, through the variety of samples and their relative complexity, far-field scanning implementation remains the most versatile approach. The implementation previously detailed in Figure 11 was considered for the initial integration of the newly introduced FMCW transceiver. Accounting for the high available potential measurement rate, a 30 min recording time is required for a 15 × 15 cm surface area scan with a 1 mm pitch; hence, we used a 150 × 150 recording grid, highly restricted by the displacement speed of the translation stages. The radar can take up to 7629 measurements per second; therefore, without being restricted by the translation stages, it can take the 150 × 150 measurement in 3 s. Through this initial transitional setup, the inspection results, gathered on a selection of samples, will highlight the global capabilities of the developed 150 GHz transceiver for volumetric inspection with the consideration of few very pragmatic and practical problems encountered by industrial actors.

### 3.1. Polymer Injected Sample Monitoring

To begin, a polymer cube facet was chosen as it incorporates features suitable to illustrate different aspects of the scanner system, such as longitudinal inspection capabilities, resolution, and dynamic range. As a large majority of polymers, this sample remains suitable for millimeter-wave inspection thanks to its high transparency.

**Figure 12:**
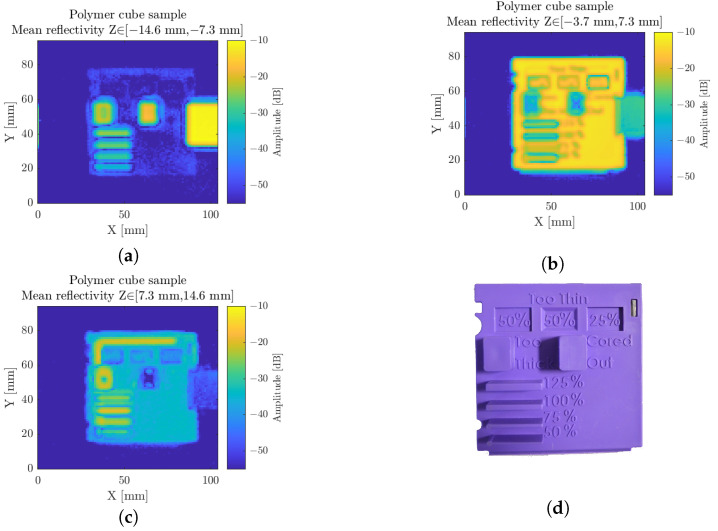
The polymer cube scan, featuring several geometrical test structures, depicted in different longitudinal ranges of interest. Namely, (**a**) is centered on the foreground protrusions, (**b**) is on the main sample plane, (**c**) is over the background features. (**d**) Photography of the sample.

A resolution of 2 mm for an NA = 0.5 configuration is achieved through a typical focused point raster scan scheme. Over such a simplistic multiplane sample, at terahertz wavelength, a large amount of information is gathered from the FMCW inspection. Figure 12 perfectly describes the depth-sensing capabilities of the transceiver with the respective consideration of each sample main plane. The first plane, in Figure 12a, features the foreground elements’ first interface and the sample holder, elevated from roughly 11 mm with respect to the main sample plane. The main sample plane itself is depicted in Figure 12b with the visualization of the various inscriptions featured on the front face, while the background, displayed in Figure 12c, features the back polymer ridge on the top of the sample, as well as the backside of the thick cubic polymer protrusions, and parallel stripes. Due to the larger optical thickness crossing through those components, their back interfaces appear further away than their actual position. This additional optical propagation delay induces a drastic contrast between the two hollow-cored and the polymer-filled cubic protrusions in this representation.

Considering the maximal reflectivity image, once again, a pseudo-topographic map of the sample could be collected. From those preliminary visualization results on a complex shape sample, several very pragmatic problems are already addressed with dimensioning topics: in the lateral and longitudinal directions, fault detection with the detection and spatial estimation of inclusions, bubbles, and lack of matter for injected samples, cracks, or delaminations, as well as homogeneity inspection.

### 3.2. Polymer Gluing

**Figure 13:**
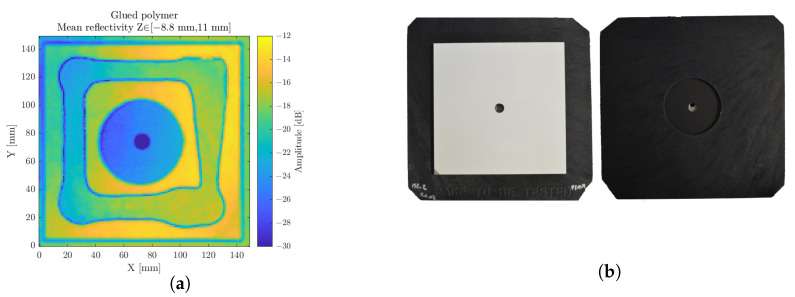
The glued polymer scan, considered on a longitudinal range of interest (**a**). Photography of the front and back sides of the sample (**b**).

Besides structural monitoring, such penetrating inspection can deliver significant information for the assessment of joints and junctions on polymers or composite dielectric materials, as long as their absorption levels allow it. Namely, the evaluation of the homogeneity and regularity of the glue or welding traces is of interest to generate a corrective feedback on production lines, hence limiting the excessive use of active matter while ensuring the integrity of the component. Namely, the expected mechanical properties of welds are at stake, along with the joints hermeticity, which represents a challenging topic ranging from the field of pharmaceutical components, such as blood bags, down to the food industry. Figure 13 displays the scan over the central portion of two 5 mm thick polymer plates glued together, inducing a 2 mm interstice. The refractive index difference within the gap is noticed through the lowered interfaces reflectivity in the presence of glue with respect to the polymer–air interfaces.

### 3.3. Chocolate Bar

The food industry is an other application field for NDT purposes, where a drastic issue is to find foreign bodies within products. Due to the strict policies imputed by the legislation, common scanning techniques such as X-ray are forbidden. Fortunately, terahertz waves do not affect the matter they pass through; we refer to this as nondestructive testing. Working at a distance, with adequate penetration depth and without impacting food products in accordance with food legislation, makes millimeter-wave radar a proper candidate for such NDT approaches [26,27,28].

**Figure 14:**
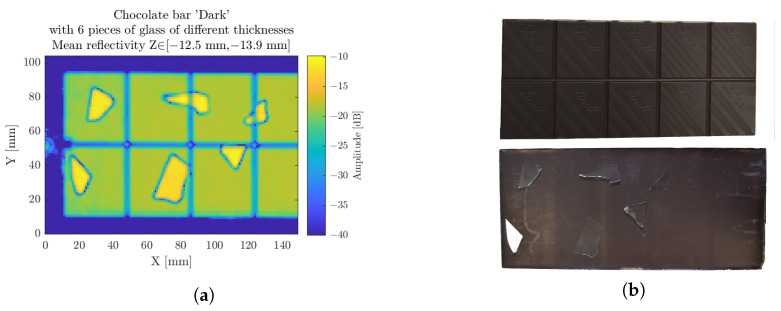
The chocolate bar scan, considered on a longitudinal range of interest on the backside (**a**). Photography of the front and back sides of the sample (**b**).

Figure 14 features the scan of a chocolate bar with some thin broken glass pieces, typically simulating the explosion of a light bulb, or protective window, on a production site. The radar can locate the position of the glass, here on the backside, and show its size. A contrast of 8 dB is witnessed between the reflection of the glass pieces and the reflection of the backside of the chocolate bar. Hence, even thinner and smaller glass pieces are located.

**Figure 15:**
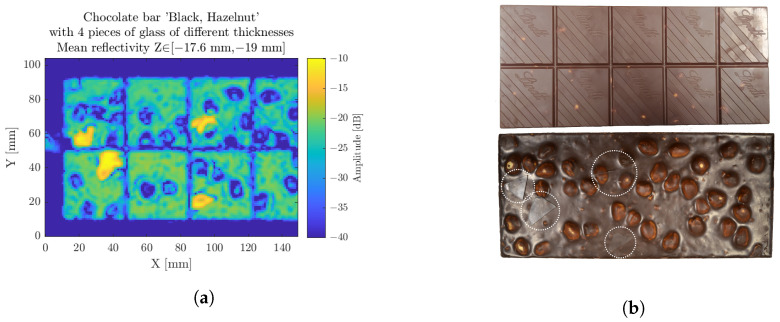
The chocolate hazelnut bar scan, considered on a longitudinal range of interest on the backside (**a**). Photography of the front and back sides of the sample (**b**).

Figure 15 exhibits a scan of another chocolate bar, featuring hazelnuts this time. Similar to the previous one, the glass pieces are easily located and there is a 10 dB difference of reflection between the glass pieces and the chocolate bar backside.

### 3.4. Crushed Granite Cylinder

In order to demonstrate the penetration capacity of radar in different types of materials, granite is an interesting candidate. It opens the bridge with its many applications in the construction sector. Millimeter-wave radar technology is able to locate cracks in a wall, as well as their sizes, preventing the degradation of buildings or monuments [29]—prevention is better than cure. The radar can also locate a demarcation between two materials of different indexes, which is linked to the density, and draw a map of the density within the sample.

**Figure 16:**
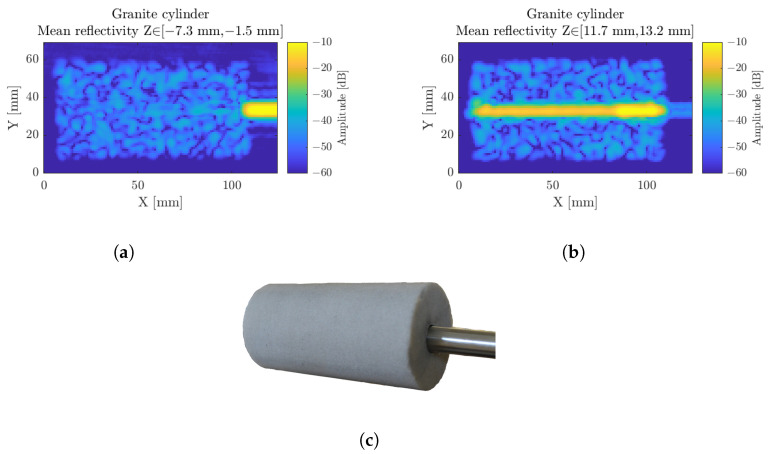
The crushed granite cylinder, considered on a longitudinal range of interest, on the top side (**a**) and on the center (**b**). Photography of the sample (**c**).

Figure 16 displays the scan of a crushed granite cylinder. In Figure 16a, the reconstruction is considered on a longitudinal range inside the top part of the cylinder. A speckle-like pattern is recorded due to the diffusive characteristic of the material, leading to random interference, picked up by the radar. A careful investigation of those patterns nevertheless enables the detection of cracks and other inhomogeneities and defects. Figure 16b displays the center of the cylinder, showing, on the right, the metal rod inside the cylinder and, on the left, the center hole. The metal rod in Figure 16a,b does not appear on the same depth because it takes less time for the wave to pass through the air than the granite cylinder, due to its higher index.

### 3.5. Pharmaceutical Packaging Assessment

To demonstrate one more time the versatility and wide application fields reachable through this homodyne sensing scheme, a pharmaceutical packaging inspection was successfully undertaken, with the simultaneous inspection of the leaflet presence and pills count. In this specific scope of application, the weakly interacting nature of millimeter waves once again offers a strong argument to ensure the integrity of the chemical compounds after inspection. Indeed, in the strictly controlled pharmaceutical industry, radiography testing remains proscribed, as an alteration of the chemical compound integrity is to be feared, along with the thermal effect that could be induced by thermography inspections.

**Figure 17:**
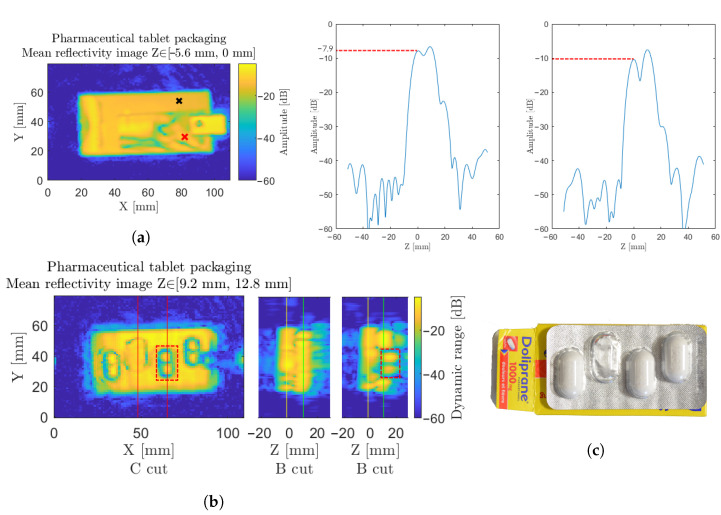
(**a**) Pharmaceutical sample under inspection, considered on a longitudinal range of interest, for the front packaging and instruction leaflet. The red cross corresponds to the leaflet and is drawn on the plot in the middle, and the black cross corresponds to the nonexistent leaflet drawn on the right. (**b**) The tablets in their blister pack, completed by two B cuts along the vertical lines, with red markers at x = 50 mm and x = 65 mm. Yellow and green markers indicate the cardboard interface and the blister pack metallic interface, respectively, on the B cuts. One pill is highlighted through the orange dotted box on both cuts. (**c**) Photography of the pharmaceutical sample.

In Figure 17a, at first glance, in the longitudinal range corresponding to the front of the packaging cardboard box, the inspection of the sealing packaging flap is conducted along with the determination of the leaflet presence. This plane corresponds to the yellow marker on the two B cuts. Moreover, in Figure 17a, two points on the image are shown. The difference between both lies in the amplitude of the first peak, related to the first interface, which emanates from the contributions of the cardboard packaging convoluted with the contributions of the leaflet. A lowered reflectivity is expected and is noticeable in the presence of the leaflet. Standard methods, such as imaging edge detection and image recognition, can hence be used to detect the presence, or not, of the leaflet inside the pharmaceutical package.

Additionally, within the sample, beyond the cardboard packaging, in Figure 17b, the pill count is easily identified through the protection blister. This visualization is performed when considering the contribution of the tablet blister pack, relative to the green markers on the B cuts. Counterintuitively, due to the high transparency and low refractive index of the pills themselves, the signal witnessed over the tablet does not emerge from a direct reflection of the pills, but relates to the reflection on the back-metallic sheet, propagated back and forth through the pill. The additional propagation delay can indeed be noticed on the second B cut on the right of Figure 17b in the orange squared section. In the presence of a pill, the metallic sheet interface appears further away than in the absence of a pellet. The refraction on the rounded edges of the pill nevertheless ensure a reliable pellet boundary detection, as detailed in the orange squared section of the C cut of Figure 17b.

## 4. Conclusions

Following the developments in the continuous wave mode of operation, for full-field and focused point sensing, the progression toward coherent sensing was achieved through the depicted advancements on a millimeter-wave homodyne FMCW transceiver. Driven by the settled capabilities of such tools, this transition hinged on the technical upgrade of a high-power Schottky-based tunable source, along with the establishment of the frequency modulation sensing mode of operation. Namely, this alteration lies in the development of a 150 GHz monostatic reflection module featuring a harmonic mixing detection scheme.

Following such a targeted integration in the pursuit of further applied proof-of-work developments, an assessment of the capabilities of this FMCW unit was carried out. A variety of considerations, specific to the FMCW sensing method, brought up an extensive and quantitative performance evaluation. Namely, the theoretical longitudinal resolution of 4.6 mm was confirmed, with a reduction down to 4 mm at −3 dB when considering a $sinc^{2}$ apodization window, or an effective resolution of 7.4 mm for high dynamic measurement with the application of the high-dynamic-range Blackman window. Precision levels down to 3.6 μm and a relative accuracy level of 110 μm were assessed for the transceiver in typical sensing configurations. This was accompanied by the achievable measurement dynamic range ranging up to 100 dB for a few seconds of integration time. As a prominent performance marker, the latter displayed a 15 dB improvement over the state-of-the-art systems featuring similar technology at similar measurement cadences. In a more system-oriented consideration, the effective measurement cadence of 7.62 kHz was successfully tested along with the long-term stability of the system.

Through the consideration of few representative samples, a glimpse of the wide, advanced problems that are accessible through millimeter-wave FMCW radars in pragmatic and specific industrial topics was provided. For such industrial applications, even at such attainable high recording cadence of 7.62 kHz, raster-scan operations remain nonviable beyond academic proof-of-work demonstrations. For a full three-dimensional contactless monitoring of the sample, a scan takes less than 15 min, but it is highly limited by the scanning unit. It was improved from several hours for a QCW two-dimensional inspection, but the transposition to the industrial sector would require full in-line inspection capabilities. At this point, the integrability of such systems does not rely on the radar mode of operation and performances, but remains tied to the imaging system capabilities. Efforts toward line-scanning setups are part of the ongoing scope of development to take full advantage of the high measurement rate while suppressing the mechanical limitations of the imaging unit integrating the newly introduced FMCW transceiver.

## Figures and Tables

**Table 1:** Apodization functions.

	Main Lobe Width @ −3 dB	Secondary Lobe Height [dB]
Rectangular	0.87 × $\delta_{f}$	−13.26
Hamming	1.28 × $\delta_{f}$	−43.59
Hann	1.42 × $\delta_{f}$	−31.47
Blackman	1.62 × $\delta_{f}$	−58.11

**Table 2:** Dynamic range for different acquisition times, with different FMCW radars.

	Dynamic Range [dB]			
This work’s radar [150 GHz]	57	77	85	90
DOTNAC radar [150 GHz] [9]	40	60	70	
Transceiver [100 GHz] [12]				60
Transceiver [200 GHz] [16]				60
Integration time	100 μs	100 ms	10 ms	5 ms

**Table 3:** Longitudinal accuracy measurements.

Theoretical Step Size	Extracted Step Size	Error	Relative Error
[μm]	[μm]	[μm]	[%]
2500	2296	204	8.2
1000	955	45	4.5
500	489	11	2.2
250	240	10	4.2
110	100	10	9.1
50	32	18	36
25	23	2	8
10	7	3	30

## Data Availability

Not applicable.

## Abbreviations

The following abbreviations are used in this manuscript:

MDPI	Multidisciplinary Digital Publishing Institute
DOAJ	Directory of Open-Access Journals
FMCW	Frequency-modulated continuous wave
UWB	Ultra-wideband
NDT	Nondestructive testing
CW	Continuous wave
QCW	Quasi-continuous wave
LO	Local oscillator
HF	High frequency
IF	Intermediate frequency
VCO	Voltage-controlled oscillator
PLL	Phase-locked loop
SMA	Sub-Miniature Version A
DR	Dynamic range
SNR	Signal-to-noise ratio
SAR	Synthetic aperture radar
RMS	Root mean square
NA	Numerical aperture
